# Atomic structures of a liquid-phase bonded metal/nitride heterointerface

**DOI:** 10.1038/srep22936

**Published:** 2016-03-10

**Authors:** Akihito Kumamoto, Naoya Shibata, Kei-ichiro Nayuki, Tetsuya Tohei, Nobuyuki Terasaki, Yoshiyuki Nagatomo, Toshiyuki Nagase, Kazuhiro Akiyama, Yoshirou Kuromitsu, Yuichi Ikuhara

**Affiliations:** 1 Institute of Engineering Innovation, School of Engineering, The University of Tokyo, Tokyo 113-8656, Japan; 3 Central Research Institute, Mitsubishi Materials Corp., Naka, Ibaraki 311-0102, Japan

## Abstract

Liquid-phase bonding is a technologically important method to fabricate high-performance metal/ceramic heterostructures used for power electronic devices. However, the atomic-scale mechanisms of how these two dissimilar crystals specifically bond at the interfaces are still not well understood. Here we analyse the atomically-resolved structure of a liquid-phase bonded heterointerface between Al alloy and AlN single crystal using aberration corrected scanning transmission electron microscopy (STEM). In addition, energy-dispersive X-ray microanalysis, using dual silicon drift X-ray detectors in STEM, was performed to analyze the local chemistry of the interface. We find that a monolayer of MgO is spontaneously formed on the AlN substrate surface and that a polarity-inverted monolayer of AlN is grown on top of it. Thus, the Al alloy is bonded with the polarity-inverted AlN monolayer, creating a complex atomic-scale layered structure, facilitating the bonding between the two dissimilar crystals during liquid-phase bonding processes. Density-functional-theory calculations confirm that the bonding stability is strongly dependent on the polarity and stacking of AlN and MgO monolayers. Understanding the spontaneous formation of layered transition structures at the heterointerface will be key in fabricating very stable Al alloy/AlN heterointerface required for high reliability power electronic devices.

Heterostructures between metals and ceramics have been widely used for power electronic devices requiring both high thermal performance and reliability in harsh environments. Since the interfaces play critical roles in many properties such as mechanical strength, thermal conductivity and dielectric strength, a fundamental understanding of the interface structure and the interface formation mechanism is crucially important. So far, several experimental and theoretical studies on metal/ceramic interfaces have been performed, down to atomistic dimensions^1^,^2^,^3^,^4^,^5^,^6^. These studies suggested that there are several factors affecting the structures of heterointerfaces, such as lattice mismatches, chemical bonding states and dopant/impurity segregation. However, one of the most important aspects when considering the formation of heterointerfaces is the bonding process^1^,^7^,^8^. Thus, in order to understand and control the heterointerface structures and their resultant properties, the actual bonding processes must be considered.

Aluminum nitride (AlN) is considered one of the most important materials for power electronic device applications due to its high thermal conductivity, low thermal expansion coefficient and nontoxic nature^9^. Metal aluminum (Al)/AlN heterostructures, fabricated by a direct bonding aluminum (DBA), are now widely used in automobiles as high power modules which can perform under harsh thermal stress conditions^10^. In DBA, the system is heated near the melting point of Al metal to facilitate liquid phase bonding between the molten Al and the AlN substrate. The melting temperature of Al metal can be decreased, via the eutectic reaction, by doping atoms such as Si into the Al metal^11^. Given the polar nature of the crystal structure, surface/interfaces of AlN crystal have received considerable attention as fundamental physics^3^,^12^. However, it is still an open question how the dopants/impurities in this system affect the bonding mechanisms of the interface at atomic dimensions and how these heterointerfaces are actually formed during the liquid bonding processes. In this work, we use aberration corrected scanning transmission electron microscopy (STEM) combined with X-ray spectroscopy to directly determine the atomic-scale structure and chemistry of the Al alloy/AlN interfaces, fabricated by the liquid phase bonding method.

In order to achieve the model liquid phase bonded Al alloy/AlN interface suitable for STEM observations, we used single crystalline AlN substrate as a base material. A commercially available epitaxial AlN film on a sapphire (0001) substrate (DOWA Electronics Materials Co. Ltd.) was used for the AlN substrate. In this substrate, an AlN single crystal was grown on sapphire with an epitaxial relationship of AlN (0001)//Al_2_O_3_ (0001), AlN 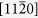//Al_2_O_3_ 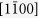, as in similar deposition techniques^13^,^14^. While the AlN polarity on the sapphire basal plane may be variable depending on the growth conditions, we directly confirmed by atomic-resolution STEM that the top surface of the present AlN substrate was an Al-polar surface. The substrate was exposed to air during the sample handling, however no surface treatment was performed before bonding.

In this study, alloy A6063 specified in Japan Industrial Standards (JIS) was selected as the model Al alloy for bonding with the AlN (0001) substrate. According to JIS, Mg and Si are the main dopants in this alloy. While the addition of Mg and Si to Al metal has a well-known function of improving mechanical properties such as work hardening, these dopant elements also lower the melting temperature of Al via the eutectic reaction. We examined the Al alloy using ICP analysis (SPS3100, SII NanoTechnology Inc.) and atomic percentages of 0.42% Mg and 0.38% Si were measured. In addition, we found other impurity elements in this metal such as 0.35% Fe, 71 ppm Cu, 589 ppm Mn, 19 ppm Cr, 73 ppm Zn, and 195 ppm Ti. Interestingly, these impurity elements did not actually contribute to the formation of the interface structure as we will discuss later. For the liquid phase bonding, Al alloy was sandwiched between the two single crystalline AlN substrates as schematically shown in Fig. 1a. The sandwiched sample was heated in vacuum at 650 °C for 0.5 h. During the heat treatment, the Al-alloy was fully melted and well wetted on the AlN surfaces. For the (S)TEM sample preparation, a standard procedure using ion thinning was used. For atomic-resolution STEM observations, we used an aberration-corrected STEM equipped with dual silicon drift detectors for energy-dispersive X-ray spectroscopy (EDS), operated at 200 kV.

## Results

**Figure 1:**
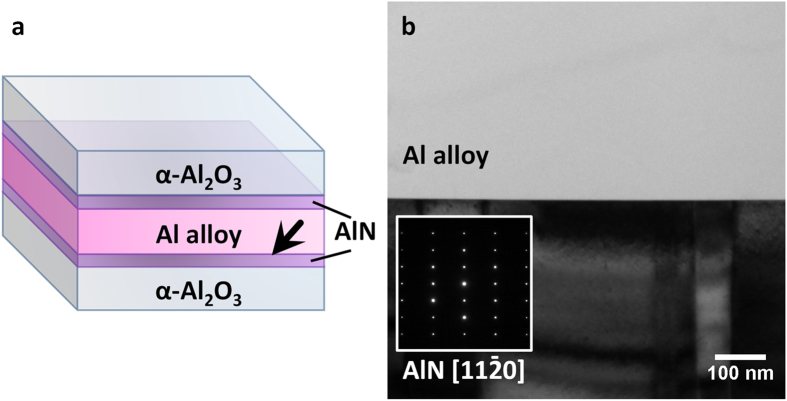
Liquid-phase bonding materials for (S)TEM examinations. (**a**) Schematic illustration of the Al alloy/AlN interfaces fabricated in this study. (**b**) A cross-sectional TEM image of the Al alloy/AlN interface. The inset SAED pattern shows that AlN is observed along 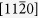 direction.

Figure 1b shows a low-magnification cross-sectional bright-field transmission electron microscopic (TEM) image of the Al alloy/AlN interface fabricated in this study. A selected area electron diffraction (SAED) pattern taken from the AlN single crystal region (inset image) indicates the 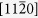 zone axis of AlN. The image contrast of AlN varied with several hundred nanometer intervals due to slightly tilted single columnar domains known as the mosaic structure^15^,^16^. Since the interface appears to be flat with no gaps and/or pores, and it is assumed that significant morphological changes between Al alloy and AlN crystal did not occur during the bonding processes, while a large step formation was found in an Al/Al_2_O_3_ interface with the same bonding method^11^. There appeared to be no preferred crystallographic orientation between the Al alloy and AlN single crystal (Figure S1). After the observation of the interface regions, sub-micron octahedral grains of spinel (MgAl_2_O_4_) within the Al alloy regions, and Mg-silicide (Mg_2_Si) grains as interfacial precipitates were occasionally present. This indicates that Mg and Si may tend to concentrate at the interface regions. However, the dominant feature of the heterointerface is the flat bonding interface regions between the Al alloy and the AlN single crystal substrate as shown in Fig. 1b.

**Figure 2:**
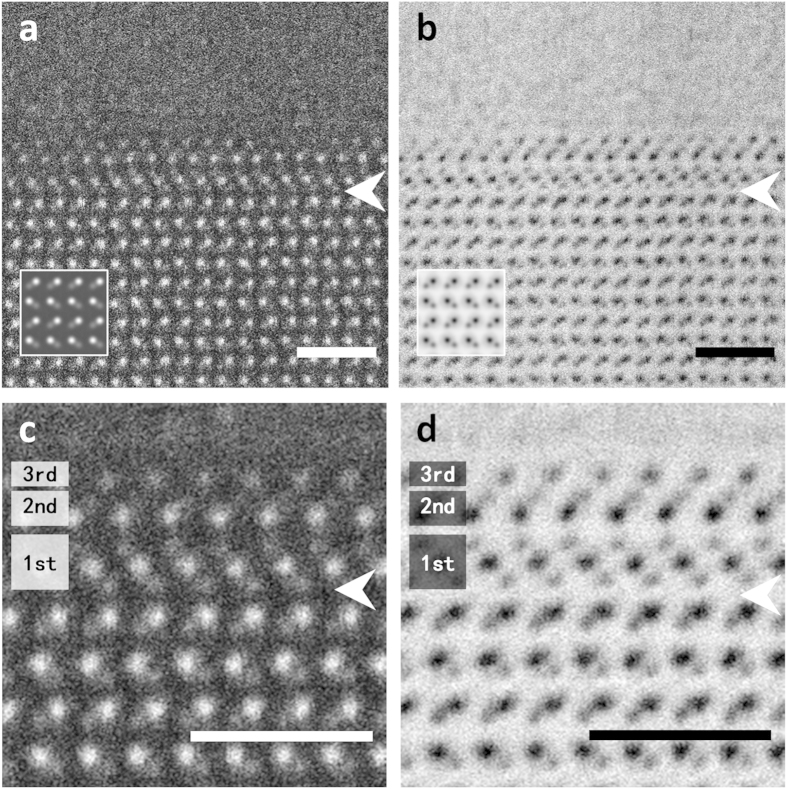
STEM images for the interface at atomic resolution. Simultaneously obtained atomic-resolution HAADF (**a**) and ABF (**b**) STEM images of an Al alloy/AlN interface. Calculated images of AlN bulk are superimposed in the lower left in a and b. The magnified views of the averaged HAADF (**c**) and ABF (**d**) STEM around the interface region indicated by the white arrows. Three types of layered structures are found above the AlN substrate shown as three bands. Each image is on its own minimum to maximum scale. All scale bars correspond to 1 nm.

Figure 2 shows the cross-sectional (a) high angle annular dark field (HAADF) and (b) annular bright field (ABF) STEM images of the Al alloy/AlN interface observed simultaneously. In both images, the atomic structure of AlN observed from 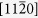 was clearly resolved. In the AlN bulk region, local dark intensity in the ABF image corresponds to the position of Al or N columns. Comparing with the HAADF image, where heavier columns have brighter intensities, the columns with weaker intensities in the ABF image correspond to the anion N columns. These images are in good agreement the simulated images, shown in the lower left-side insets, which means that the AlN substrate has an Al-polar growth direction in the present examination. On the other hand, the atomic structures of the Al alloy region were not clearly resolved because the present viewing direction is not well aligned along the certain high symmetry crystallographic axis (Figure S1). Therefore, in these images, the interface atomic structure of only the AlN side can be identified. However, on the AlN side, we see that the atomic structure at the interface region (see the white arrows in Fig. 2) appears to deviate from the crystal structure of AlN. The types of atomic columns-whether they are anion (lighter element; O or N) or cation (heaver element; metals) columns-can be determined from the image intensity differences. To highlight the differences clearly, we show noise-filtered images of the interface core region in Fig. 2c,d. From these images, we may divide the interface core region into three different layered structures labelled as the 1^st^, 2^nd^, and 3^rd^ layers. Considering both HAADF and ABF STEM images, the 1^st^, 2^nd^ and 3^rd^ layer interface structures are anion-cation-anion, cation-anion and anion layers, respectively. These layers are fully coherent with respect to the AlN bulk structure, suggesting that the growth of the three-layered structures is strongly affected by the structure of the AlN substrate. Moreover, the 1^st^ and 2^nd^ layers appear to consist of octahedral and tetrahedral coordination polyhedra, respectively. Similar stacking atomic arrangements of [Al(O,N)_4_] tetrahedra and [Al(O,N)_6_] octahedra have been recently suggested as the stable structure of aluminum oxynitride (AlON)^17^. However, it is difficult to determine the detailed atomic structure of the three layers from the STEM image contrast since dopant elements such as Al and Mg with close atomic numbers may coexist. Thus, we performed atomic-resolution chemical mapping using STEM-EDS.

**Figure 3:**
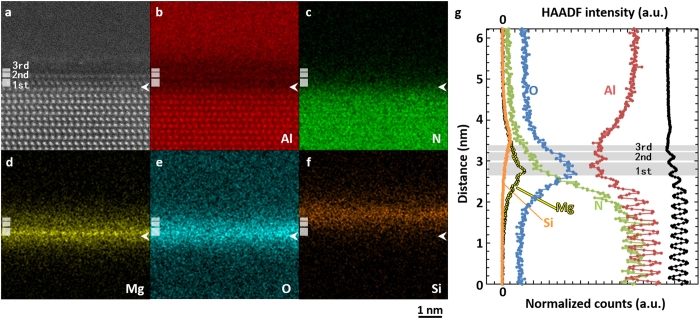
Atomic-scale elemental maps of the Al alloy/AlN interface. HAADF STEM image (**a**) and corresponding elemental maps of Al (**b**), N (**c**), Mg (**d**), O (**e**) and Si (**f**) are shown. The positions of 1^st^, 2^nd^ and 3^rd^ bands and the white arrows coincide with those in Fig. 2. The intensity line profiles (summed along the interface direction) of the HAADF and of each elemental maps are shown in (**g**).

**Figure 4:**
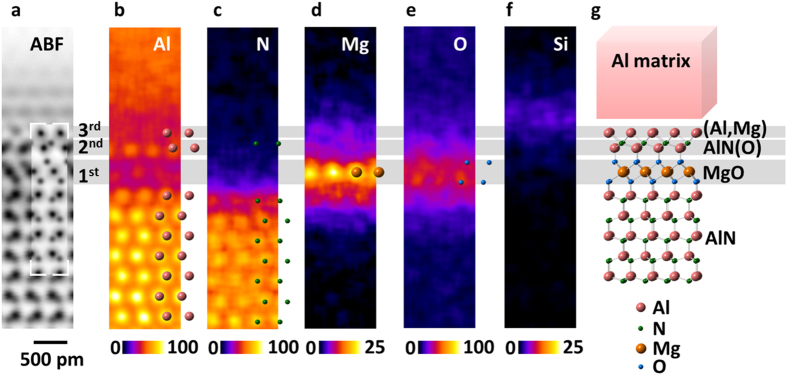
Atomic-scale structural determination of the Al alloy/AlN interface. The averaged ABF-STEM image (**a**) and the corresponding elemental maps of Al (**b**), N (**c**), Mg (**d**), O (**e**) and Si (**f**) were obtained by summing 56 SIs, respectively. The structure model of the heterointerface (**g**) determined from the experimental results is shown. The color bar shows the X-ray intensity, normalized by corresponding *k*-factors.

Figure 3 shows atomically resolved chemical maps of the Al alloy/AlN interface using STEM-EDS. The elemental maps of Al, N, O, Mg and Si, are shown. In the present results, Mg was the only foreign metallic element, which contributes to the formation of the interface layered structures. Line profiles of HAADF and corresponding X-ray signals are shown as Fig. 3g. X-ray signal profiles shown here are converted into normalized counts (which is related to the number of atoms) using Cliff-Lorimer *k*-factors^18^,^19^, although quantitative compositional information on that atomic scale requires one to more carefully treat the effects of channeling^20^. We found that the highest signal in Mg and O maps is located at the 1^st^ interfacial layer, but that of Si is slightly shifted within Al alloy region. This indicates that these elements should occupy different atomic layers at the interface region. Broad signal of Si and very weak signal of Fe (not shown here) are found above the 3rd layer (in Fig. 4). This suggests that Si and Fe atoms are segregated to the Al alloy side of the interface rather than forming a silicon or iron oxide layer (See supplementary information for the Fe chemical map, shown as Figs 2 and 3). This means that Si and Fe did not directly contribute the formation of the interface-layered structure. In the 1st layer, Mg atoms are concentrated to a single atomic column layer, whereas O atoms are concentrated to the top and bottom of the Mg layer. Considering the HAADF/ABF STEM images shown in Fig. 2, the cation and anion columns in the 1st layer can be thus identified to be Mg and O atomic columns, respectively. Considering the bonding distances and angles between Mg and O columns in the 1st layer, this structure is very similar to MgO_6_ octahedron with rocksalt structure. Thus, the 1st layer can be identified to be the MgO_6_ octahedron monolayer, whose orientation relationship to the AlN substrate is MgO (111)//AlN (0001), MgO 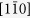//AlN 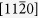 with the lattice mismatch of −4.2%. In the 2nd layer, a local maximum of Al can be found at the cation columns. Thus, the cation columns can be identified as Al columns. O and N could not be separated in the 2nd layer, indicating some intermixing may be occurring in the anion layer. The image contrast of the 2nd layer is similar to the Al-N contrast in the AlN bulk structure, although with an inverted polarity. This image contrast cannot be assigned to the several possible structures based on aluminum oxides. Thus, we consider that the main structure of the 2nd layer is an AlN_4_ tetrahedral monolayer. The polarity of this layer is inverted from the AlN substrate due to the presence of the MgO interlayer. The structural analysis using atomic EDS mapping is summarized in Fig. 4; local maximum contrast of each map is consistent with the structure model. From the 3rd layer, Al starts to continuously increase toward the Al alloy region. This suggests that atoms located at the cation site in the 3rd layer are mainly Al. Si and Fe atoms are segregated to the Al metal side of the heterointerface. We note that the amounts of Si and Fe at the interface region are too small to form distinct interface layer structures like Mg (Figure S4).

**Figure 5:**
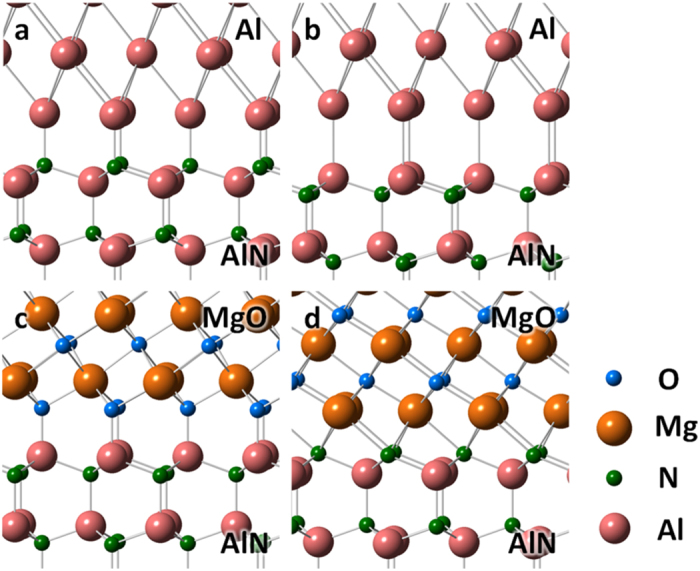
The energetically stable DFT models for heterointerfaces of different polarity. Al/AlN (**a**) and MgO/AlN (**c**) compared with those with inversion polar (**b**,**d**), respectively.

In order to verify that the formation of inverted AlN layer at the interface is energetically possible, we performed DFT calculations of several Al/AlN hetero interface models. To compare adhesion energy (or work of separation) of Al/AlN interface for different AlN polarities (N-polar and Al-polar), simple models for the coherent interfaces are considered: we constructed supercells containing Al and AlN crystal slabs and vacuum layer(s) with facing Al (111) plane to AlN (0001) planes with different polarities. Adhesion energy of an interface is obtained as the difference between a cell energy with two slabs (Al and AlN) separated and a cell energy with two slabs attached to form one Al/AlN interface. By defining the energy of isolated slabs of Al, AlN, and an attached slab of Al/AlN as ε_*Al*_, ε_*AlN*_ and ε_*Al/AlN*_, respectively, the adhesion energy can be expressed as *E*_*ad*_ = ε_*Al*_ + ε _*AlN*_ − ε _*Al/AlN*_. The estimated adhesion energy *E*_*ad*_ of N-polar and Al-polar interfaces were 4.45 J/m^2^ and 2.46 J/m^2^, respectively (Table 1). These results indicate that the Al/AlN interface structure with an N-polar structure is energetically more favorable than that of an Al-polar structure. Additionally, the adhesion energy of AlN/MgO interfaces for different MgO {111} polarities (O-polar and Mg-polar) were also obtained in a similar manner. Calculated results of adhesion energy for Al/AlN and AlN/MgO interfaces are shown in Table 1. Figure 5 shows relaxed atomic structures of these interface models. Although the Al/AlN interface favors the N-polar structure shown as Fig. 5a, the AlN/MgO interface favors the Al-polar AlN with O-polar MgO shown as Fig. 5c – i.e., it favors strong Al-O bonding due to its large difference in electronegativity. The interfacial bonding distance shows a good correlation with the adhesion energy as shown in Table 1. We found that the Al-O bonded AlN/MgO interface is the most stable in terms of adhesion energy among these interface structures considered. These results are consistent with our observation that, although the surface of AlN substrate is originally Al-polar, the monolayer of N-polar AlN structure is formed on top of the bonding surface by inserting the MgO monolayer to facilitate the interface bonding between Al alloy and AlN.

## Discussion

Very complex, atomic-scale layered interface structures are formed at the interface between Al alloy and AlN crystal after liquid phase bonding processes due to the presence of Mg. Mg in Al alloy has a tendency to segregate to the metal surface or grain boundary^21^,^22^,^23^ and the MgO formation due to native alumina (Al_2_O_3_) on surfaces of Al alloy or AlN before bonding.

Here, we discuss the thermodynamic stability of MgO phase at the interface region. When Al alloy (containing Mg) is melted on AlN substrate, a reaction between Mg and surface oxide (Al_2_O_3_) of AlN and/or Al alloy may occur. Since Al_2_O_3_ becomes an oxidizing agent for Mg in this system, a spinel structure should be initially formed by the following reaction^24^;


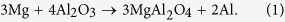


If metallic Mg is continuously provided from the Al alloy, a subsequent reaction between Mg and MgAl_2_O_4_ may occur under reducing conditions. Thermodynamically, with the possible existences of metal Mg, MgO becomes more stable and the following reaction can take place;


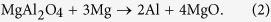


A previous experimental report demonstrated that the above reaction is able to take place by using thermal reaction couples of molten Mg and 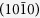 α-Al_2_O_3_ surface^25^. Their X-ray pole figure analysis showed some particular crystallographic orientation between MgO and α-Al_2_O_3_ as a result of incongruent reduction; Al_2_O_3_ is decomposed into Al metal and O and thus Mg is finally incorporated as MgO. Similar reactions have been also suggested in recent literature, exhibiting the structure of double layer of MgO crystallite/amorphous Al_x_O_y_ found in the Al-Mg alloy/ceramics composite material^26^. Although these reactions require the presence of Al_2_O_3_ surface film on the AlN surface, our preliminary observation confirmed the presence of oxygen on surface of the AlN substrate used in this study. Stable existence of the surface oxide of AlN has been also reported^27^,^28^ and may be unavoidable even handled with care at room temperature^29^.

Since MgO is strongly ionic, a flat MgO (111) free surface is likely to be unstable^30^,^31^,^32^. Nevertheless, our results indicate the formation of an O-Mg-O stacking MgO (111) layer. Neither reconstruction on the rocksalt (111) surface geometry nor micro-faceting of the {100} nonpolar planes are observed. On the other hand, the {111} surface planes are dipolar and thus a charged surface should have a divergent electrostatic energy. An early report using TEM observations by Mader and Maier showed the precipitation of octahedron MgO has the polar {111} facet planes in high purity (N5) noble metals^33^. They expected that a sharply terminated MgO {111} plane should have a net charge; the net charge induces a compensating image charge in the metal and this effect may strengthen the bonding^3^,^34^. The rocksalt oxide will have no residual dipole moment, but it would be non-stoichiometric and charged when a polar-terminated surface is exposed to outside. By using an ultrahigh-vacuum (UHV) system, an epitaxial growth of atomically flat MgO (111) on Ag (111) has been performed up to 10-ML in thickness, where image charge is induced in the bulk metal^35^. In the present case, the rocksalt MgO (111) layer is sandwiched in between an Al-polar AlN layer, which may effectively compensate such surface extra charges. Further theoretical analysis is needed to fully understand such stabilization mechanisms. However, spontaneous formation of MgO atomic layer should facilitate the formation of stable Al/AlN interface by reversing the polarity of AlN. In other ceramic systems such as Si_3_N_4_, dopant effects are also known to be very important for controlling interfaces and their dynamics during liquid-phase sintering. Especially, rare-earth elements are found to preferentially occupy specific interface sites at amorphous/grain interfaces^36^,^37^,^38^ to control grain growth and their resultant mechanical properties. In our case, Mg atoms are shown to have critical effects on the interface formation in Al/AlN interfaces. These results may imply that, for the liquid phase bonding of ceramic materials, dopant atoms could be one of the crucial factors to control and engineer heterostructures. It would be thus quite important to understand the very local chemistry of heterointerfaces as shown in this study, in order to truly understand the interface formation mechanisms in ceramics.

In summary, we directly observed the atomic-scale structure and chemistry of a liquid-phase bonded Al/AlN heterointerface using aberration-corrected STEM. We found that a monolayer of MgO is formed at the interface core region, and a polarity-inversed AlN monolayer (N-polar) is formed on top of the MgO monolayer which bonds with the Al alloy region. DFT calculations confirm that the interfacial energy between Al, AlN and MgO is strongly dependent on the stacking and polarity of AlN and MgO. Consistent with our observations, we also found that N-polar AlN surface is more stable than Al-polar AlN surface to form interface with Al metal, and Al-polar AlN surface is more stable to form interface with O-polar surface of MgO. Our results thus suggest that spontaneous formation of such layered transition structures should be the key to form very stable Al alloy/AlN heterointerface by the liquid phase bonding employed in this study.

## Methods

### TEM specimen preparation

Several slices were cut from the bonding block for the edge-on interface TEM observations. The slices were ionically polished up to finale thinned by a cross-sectional Ar milling method with ion gun voltages of 1.5–5 keV and swing beam of +/−1.5deg to make an electron transparent specimen. The Ar ion milling was performed with the following procedures: we cut the bulk bonded sample in small pieces and polished it down to a thickness of 100 μm by mechanical polishing. This sample was then Ar ion milled (5 kV) down to 10 μm using an ion slicer (JEOL Ltd.). The specimen was then carefully thinned from the AlN crystalline side to minimize preferential ion-milling to the Al alloy region until perforation (see Supplemental Information for thickness of interface in Figure S6). Finally, we removed the surface damage layers by lowering the Ar accelerating voltage to 1.5 keV. More information about this method can be found in a literature^39^. The interface was observed from AlN 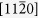 direction and then the interface is no incline from this direction.

### (S)TEM observation

TEM images, selected area electron diffraction (SAED) and EDS were used to identify phases and the composition of both the bulk and the interface. An atomic-resolved STEM-EDS mapping of the cross-sectional interface was performed using a probe forming aberration corrected STEM (JEM-ARM200F, JEOL Co., Ltd.), which is equipped with a cold field emission gun (CFEG) and a dual silicon drift detector (dual-SDD) having 200 mm^2^ of total detector area. Using SDD on atomic STEM has benefits for visualizing light elements, since low-energy peak such as N K edge (0.392 keV) is much more identifiable due to the improvement of energy resolution as compared with the conventional Si (Li) detector. Furthermore, the large detector area (solid angle of 1.7 sr) and large pole-peace gap around the TEM specimen is advantageous since it prevents electron damaged of the specimen during EDS acquisition. The scanning speed was 1 s/frame where one frame is 256 × 256 pixels. This corresponds to a pixel dwell time of 15–30 us/pixel, with a drift correlation interval of 1–4 s (1–2 frames per correction) by using a commercial EDS analyzer (Noran system 7, Thermo Fisher Scientific, Co. Ltd.). The final spectrum image (SI) map is an average of approximately 1,800 frames. X-ray peak separation analyses was carried out on commercial software (NSS3, Thermo Fisher Scientific, Co. Ltd.) using peak shape references of each element obtained on the same STEM in advance. Prior to EDS mapping, two detectors having detection angle of 11–22 mrad and 90–370 mrad were set to simultaneously obtain ABF and HAADF imaging, respectively, with probe forming aperture half-angle of 22 mrad. The HAADF/ABF image simulation was performed using commercial multislice simulation software (HREM Research Inc., xHREM). For Al, Mg, Si and Fe, we used theoretical *k*-factors with consideration for ionization cross-sections and the detector geometry^18^. For N and O, we experimentally obtained *k*-factors from thin foil specimens of AlN bulk and SiO_2_ amorphous, respectively^19^. Since the specimen thickness is approximately 30 nm for our STEM-EDS mapping, we did not perform ZAF correction throughout this study.

### Noise reductions for atomic resolution imaging and mapping

Since the interface has a periodic repeating unit structure (along the interface), we obtained high signal-to-noise ratio (SNR) unit structure images by summing many unit structure images along the interface. To further enhance the SNR in our EDS map, 14 sets of original SIs and corresponding averaged ABF-STEM images were obtained by multiple scanning. Acquisition time of 50 s is used for each set. The 14 sets of original SIs can be divided into 56 SIs because each original SI contains four repeating unit structures of the interface. After acquisitions, we corrected spatial drift in the 56 SIs with reference to the averaged ABF image. The 56 SIs were summed for each of the 2048 channels. Using this summed SI of the (averaged) single unit structure of the interface, we performed peak separation analysis and formed EDS chemical maps.

### DFT calculations

The calculations were performed using the VASP code^40^ based on density functional theory (DFT). The exchange and correlation effects were treated by the generalized gradient approximation (GGA)^41^. The cutoff energy in the plane wave expansion was 500 eV. Al-polar AlN (0001) to Al (111) and N-polar AlN 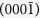 to Al (111) interfaces were modeled by assuming an Al [110]//AlN [1000], Al (111)//AlN [0001] coherent interface., We considered three translation geometries, which are determined as relative positions of the top atom of the AlN slab located under the atom of the first layer (On-top site), the second layer (Hollow1 site) and the third layer (Hollow2 site) of the Al slab at interface. We performed the same type of calculations for Al-polar AlN (0001)//O-polar MgO 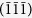, N-polar AlN 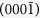//Mg-polar MgO (111), Al (111)//O-polar MgO 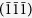, Al (111)//Mg-polar MgO (111), Mg (0001)//O-polar MgO (111) and Mg (0001)//Mg-polar MgO (111) interfaces. We constructed supercell models containing a vacuum and combination of two slabs among the following materials; AlN slab with (0001) surface (2 × 2 × 4 extension of the unit cell, composed of 64 atoms and 1.79 nm in thickness), Al slab with (111) surface (4 repeating units of the ABC stacking of the fcc along [111], composed of 48 atoms and 2.57 nm in thickness), MgO slab with (111) surface (4 repeating units of the ABC stacking of the rocksalt structure along [111], composed of 96 atoms and 2.92 nm in thickness), Mg slab with (0001) surface (2 × 2 × 6 extension of the unit cell, composed of 48 atoms and 2.87 nm in thickness), and vacuum layer(s) (about 5.0 nm in thickness) with a hexagonal cell; cell dimensions were a = b = 0.622 nm, and c = 9.0–10.0 nm (attached slab cells) or 15.0 nm (separated slab cells). In order to fit to the substrate AlN lattice (a = 0.311 nm), an expanded lattice of Al and MgO, and a contractive lattice of Mg along the interface were used to compensate for the lattice mismatch of −8.0%, −4.2% and 3.1%, respectively. k-points were sampled on a 3 × 3 × 1 mesh. All atoms within the cell were fully relaxed until residual forces on atoms became smaller than 0.05 eV/Å. Adhesion energy was determined by taking the difference of total energies of relaxed supercells with slabs separated by a vacuum layer or slabs attached to form one interface.

## Additional Information

**How to cite this article**: Kumamoto, A. *et al.* Atomic structures of a liquid-phase bonded metal/nitride heterointerface. *Sci. Rep.* **6**, 22936; doi: 10.1038/srep22936 (2016).

## Supplementary Material

Supplementary Information

## Figures and Tables

**Table 1:** Calculated adhesion energies (*E*_*ad*_) and averaged bonding distances (*D*_*int*_) of the interfaces shown in Fig. 5.

	Interface		*E*_*ad*_	*D*_*int*_
	Materials	Terminations	(J/m^2^)	(Å)
a	Al/AlN	Al-N	4.45	1.94
b	Al/AlN	Al-Al	2.46	2.71
c	MgO/AlN	O-Al	8.87	1.77
d	MgO/AlN	Mg-N	4.81	2.40

## References

[b1] Fu Q. & Wagner T. Interaction of nanostructured metal overlayers with oxide surfaces. Surface Science Reports 62, 431–498 (2007).

[b2] Alemany P. Metal-ceramic adhesion - band-structure calculations on transition-metal-AlN interfaces. Surface Science 314, 114–128 (1994).

[b3] Finnis M. W. The theory of metal-ceramic interfaces. J. Physics-Condensed Matter 8, 5811–5836 (1996).

[b4] Ernst F. Metal-oxide interfaces. Materials Science & Engineering R. 14, 97–156 (1995).

[b5] Ikuhara Y. & Pirouz P. High resolution transmission electron microscopy studies of metal/ceramics interfaces. Microscopy Research and Technique 40, 206–241 (1998). 9518055 10.1002/(SICI)1097-0029(19980201)40:3<206::AID-JEMT4>3.0.CO;2-S

[b6] Chen M. S. & Goodman D. W. Ultrathin, ordered oxide films on metal surfaces. J. Phys.: Condens. Matter 20, 264013 (2008). 21694347 10.1088/0953-8984/20/26/264013

[b7] Kaplan W. & Kauffmann Y. Structural order in liquids induced by interfaces with crystals. Annu. Rev. Mater. Res. 36, 1–48 (2006).

[b8] Oh S. H., Kauffmann Y., Scheu C., Kaplan W. D. & Ruhle M. Ordered Liquid Aluminum at the Interface with Sapphire. Science 310, 661–663 (2005). 16210498 10.1126/science.1118611

[b9] Werdecker W. & Aldinger F. Aluminum nitride – an alternative ceramic substrate for high power applications in microcircuits. IEEE Trans. Components, Hybrids, Manuf. Technol. 7, 399–404 (1984).

[b10] Kuromitsu Y. *et al.* Direct bonded aluminum on aluminum nitride substrates via a transient liquid phase and its application. *in 2010 6th International Conference on Integrated Power Electronics Systems (CIPS 2010)* (IEEE, Nuremberg/Germany, 2010).

[b11] Montesa C. M., Shibata N., Tohei T. & Ikuhara Y. TEM observation of liquid-phase bonded aluminum-silicon/aluminum nitride hetero interface. J. Mater. Sci. 46, 4392–4396 (2011).

[b12] Goniakowski J., Finocchi F. & Noguera C. Polarity of oxide surfaces and nanostructures. Rep. Prog. Phys. 71, 016501 (2008).

[b13] Tokumoto Y., Sato Y., Yamamoto T., Shibata N. & Ikuhara Y. Atomic structure of AlN/Al_2_O_3_ interfaces fabricated by pulsed-laser deposition, J. Mater Sci. 41, 2553–2557 (2006).

[b14] Kehagias Th., Komninou Ph., Nouet G., Ruterana P. & Karakostas Th. Misfit relaxation of the AlN/Al_2_O_3_ (0001) interface. Physical Review B, 64, 195329 (2001).

[b15] Qian W. *et al.* Microstructural characterization of αGaN films grown on sapphire by organometallic vapor phase epitaxy. J. Appl. Lett. 66, 1252–1254 (1995).

[b16] Tokumoto Y. *et al.* High-resolution transmission electron microscopy (HRTEM) observation of dislocation structures in AlN thin films. J. Mater. Res. 23, 2188–2194 (2008).

[b17] Asaka T., Banno H., Funahashi S., Hirosaki N. & Fukuda K. Electron density distribution and crystal structure of 27R-AlON, Al_9_O_3_N_7_. J. Solid State Chem. 204, 21–26 (2013).

[b18] Lorimer G. W. Quantitative X-ray microanalysis of thin specimens in the transmission electron microscope: a review. Mineralogical Magazine 51, 49–60 (1987).

[b19] Cliff G. & Lorimer G. W. The quantitative analysis of thin specimens. J. Microscopy 103, 203–207 (1975).

[b20] Lugg N. R., Kothleitner G., Shibata N. & Ikuhara Y. Ultramicroscopy 151, 150–159 (2015). 25535061 10.1016/j.ultramic.2014.11.029

[b21] Esposto F. J., Zhang C.-S., Norton P. R. & Timsit R. S. Segregation of mg to the surface of an al-mg single-crystal alloy and its influence on the initial oxidation at room-temperature. Sureface Sci. 302, 109–120 (1994).

[b22] Liu X. Y., Ohotnichy P. P., Adams J. B., Rohrer C. L. & Hyland R. W. Jr. Anisotropic surface segregation in Al-Mg alloys. Surface Science 373, 357–370 (1997).

[b23] Liu X. Y. & Adams J. B. Grain-boundary segregation in Al-10%Mg alloys at hot working temperatures. Acta Materialia 46, 3467–3476 (1998).

[b24] Lumley R. N., Sercombe T. B. & Schaffer G. B. Surface oxide and the role of magnesium during the sintering of aluminum. Metall. Mater. Trans. A 30A, 457–463 (1999).

[b25] Kumar P., Dregia S. A. & Sandhage K. H. Epitaxial growth of magnesia and spinel on sapphire during incongruent reduction in molten magnesium. J. Materials Research 14, 3312–3318 (1999).

[b26] Zhang X. *et al.* Metal/ceramic interface structures and segregation behavior in aluminum-based composites. Acta Materialia 95, 254–263 (2015).

[b27] Sato T., Haryu K., Endo T. & Shimada M. High-temperature oxidation of hot-pressed aluminum nitride by water-vapor. J. Mater. Sci. 22, 2277–2280 (1987).

[b28] Katnani A. D. & Papathomas K. I. Kinetics and initial stages of oxidation of aluminum nitride: Thermogravimetric analysis and x‐ray photoelectron spectroscopy study. J. vac. Sci. Technol. A 5, 1335–1340 (1987).

[b29] Huang M. R. S., Erni R. & Liu C.-P. Influence of surface oxidation on the valence electron energy-loss spectrum of wurtzite aluminum nitride. Appl. Phys. Lett. 102, 061902 (2013).

[b30] Tasker P. W. The stability of ionic crystal surfaces. J. Phys. C: Solid State Phys., 12, 4977–4984 (1979).

[b31] Henrich V. E. Thermal faceting of (110) and (111) surfaces of MgO. Surf. Sci. 57, 385–392 (1976).

[b32] Plass R., Feller M. & Gajdardziska-Josifovska M. Morphology of MgO (111) surface: artifacts associated with the faceting of polar oxide surfaces into neutral surfaces. Surf. Sci. 414, 26–37 (1998).

[b33] Mader W. & Maier B. TEM investigation of metal/MgO interfaces produced by precipitation. J. Phys. Colloques 51, C1-867–872 (1990).

[b34] Lojkowski W. & Fecht H.-J. The structure of intercrystalline interfaces. Progress in Materials Science 45, 339–568 (2000).

[b35] Kiguchi M., Entani S. & Saiki K. Atomic and electronic structure of an unreconstructed polar MgO (111) thin film on Ag (111). Phys. Rev. B 68, 115402 (2003).

[b36] Shibata N. *et al.* Observation of rare-earth segregation in silicon nitride ceramics at subnanometre dimensions. Nature 428, 730–733 (2004). 15085126 10.1038/nature02410

[b37] Ziegler A. *et al.* Interface Structure and Atomic Bonding Characteristics in Silicon Nitride Ceramics. Science 306, 1768–1770 (2004). 15576617 10.1126/science.1104173

[b38] Winkelman G. B. *et al.* Arrangement of rare-earth elements at prismatic grain boundaries in silicon nitride. Phil. Mag. Lett. 84, 755–762 (2004).

[b39] Tateno S., Sinmyo R., Hirose K. & Nishioka H. The advanced ion-milling method for preparation of thin film using ion slicer; Application to a sample recovered from diamond-anvil cell. Review of Scientific Instruments, 80, 013901 (2009). 19191441 10.1063/1.3058760

[b40] Kresse G. & furthmuller J. Efficient iterative schemes for ab initio total-energy calculations using a plane-wave basis set. Phys. Rev. B 54, 11169–11186 (1996). 10.1103/physrevb.54.11169 9984901

[b41] Perdew J. P., Burke K. & Ernzerho M. Generalized gradient approximation made simple. Phys. Rev. Lett. 77, 3865–3868 (1996). 10062328 10.1103/PhysRevLett.77.3865

