# Supplementary material for: Atomic structures of a liquid-phase bonded metal/nitride heterointerface
Source: Sci Rep. 2016 Mar 10;6:22936. doi: 10.1038/srep22936 (PMC4785497; doi:10.1038/srep22936)
Supplement: Supplementary Information [file srep22936-s1.pdf]

Supplementary Information:

Atomic structures of a liquid-phase bonded metal / nitride heterointerface

Akihito Kumamoto[1], Naoya Shibata[1,2], Kei-ichiro Nayuki[1], Tetsuya Tohei[1], Nobuyuki Terasaki[3], Yoshiyuki Nagatomo[3], Toshiyuki Nagase[3], Kazuhiro Akiyama[3], Yoshirou Kuromitsu[3] and Yuichi Ikuhara[1]*

[1]Institute of Engineering Innovation, School of Engineering, University of Tokyo, Tokyo 113-8656, Japan.

[2]Japan Science and Technology Agency, PRESTO, 4-1-8 Honcho Kawaguchi, Saitama 332-0012, Japan.

[3]Central Research Institute, Mitsubishi Materials Corp., Naka, Ibaraki 311-0102, Japan.

Supplementary Figures

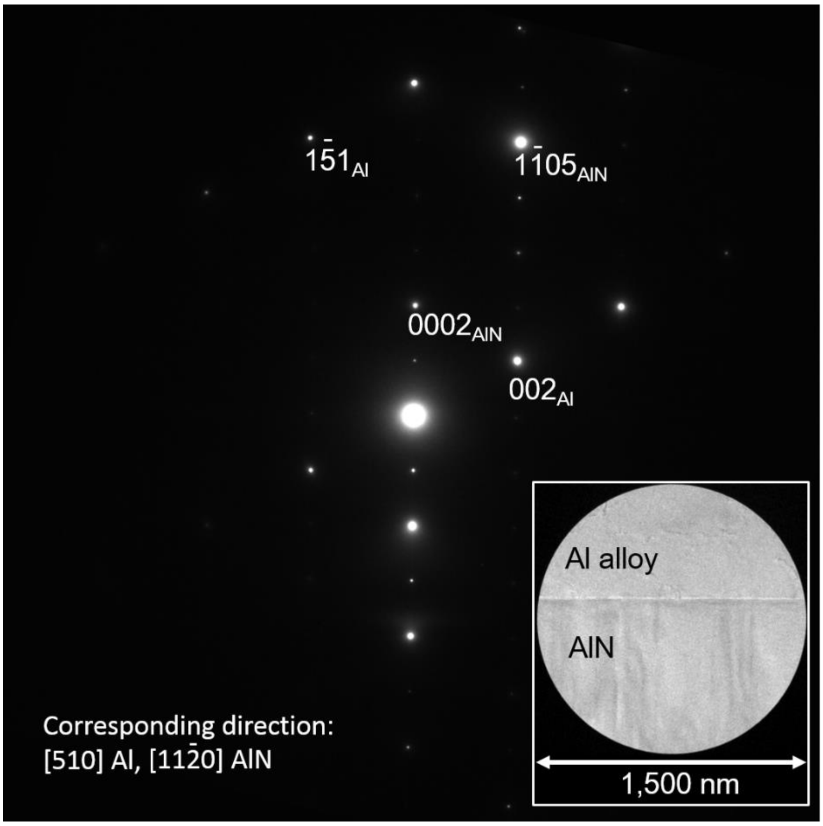

Figure S1: SAED pattern of the Al/AlN interface. Diffraction spots can be assigned to the indexes shown in the image. The observing direction is close to the [510] direction of fcc Al and [11$\bar{2}$0] direction of wurtzite AlN. The angle between Al [510] and AlN [11$\bar{2}$0] directions is about six degrees. The corresponding selected area TEM image is shown in the inset.

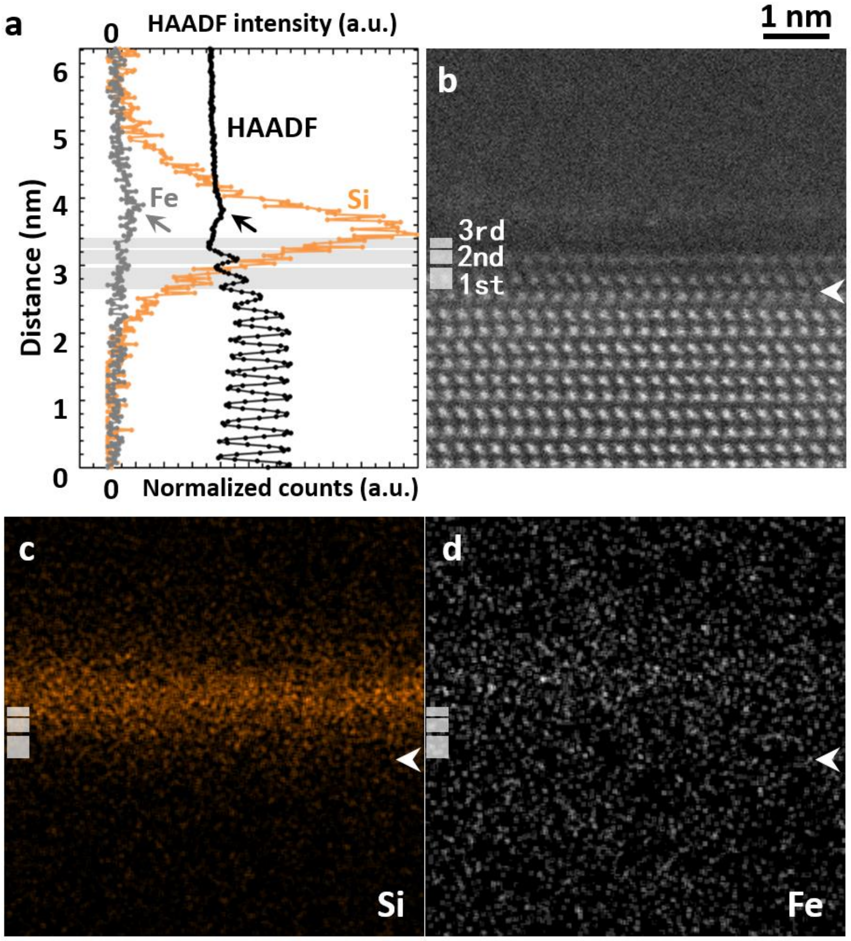

Figure S2: Atomic-scale elemental maps of Si and Fe at the Al alloy / AlN interface. (**a**) The intensity line profiles across the interface from the HAADF image, Si elemental map and Fe elemental map. Corresponding (**b**) HAADF image, (**c**) Si elemental map and (**d**) Fe elemental map, respectively. As seen in (**a**), intensity peaks of Si and Fe can be found above the 3rd layer shown in (**b**).

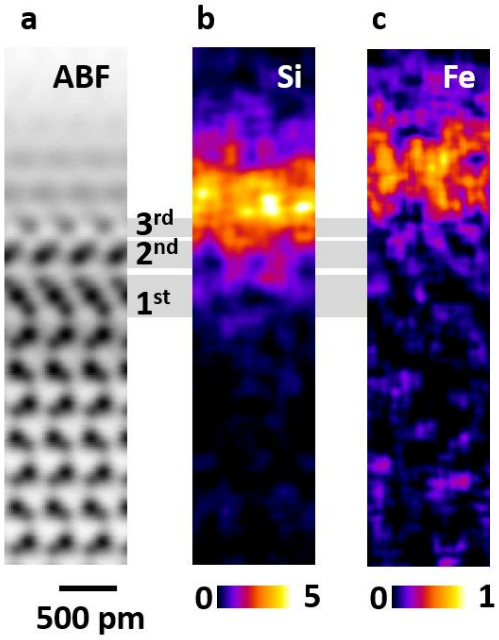

Figure S3: Higher SNR atomic-scale elemental maps of Si and Fe at the Al alloy / AlN interface. (**a**) The averaged ABF-STEM image and the corresponding (**b**) Si and (**c**) Fe elemental maps obtained by the same procedure in Fig. 4. The intensity peak of Fe segregation layer can be found slightly above that of Si segregation layer.

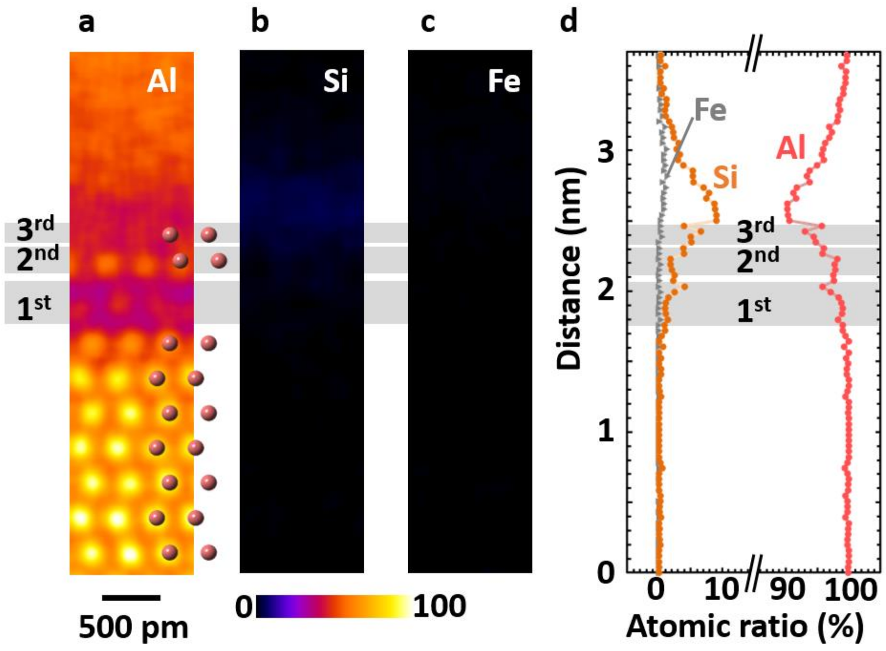

Figure S4: Comparison between Al of matrix and minor elements of Si and Fe. Al (**a**), Si (**b**) and Fe (**c**) elemental maps have the same intensity scale. Integrated line profiles of the atomic ratio of Al, Si and Fe are shown in (**d**).

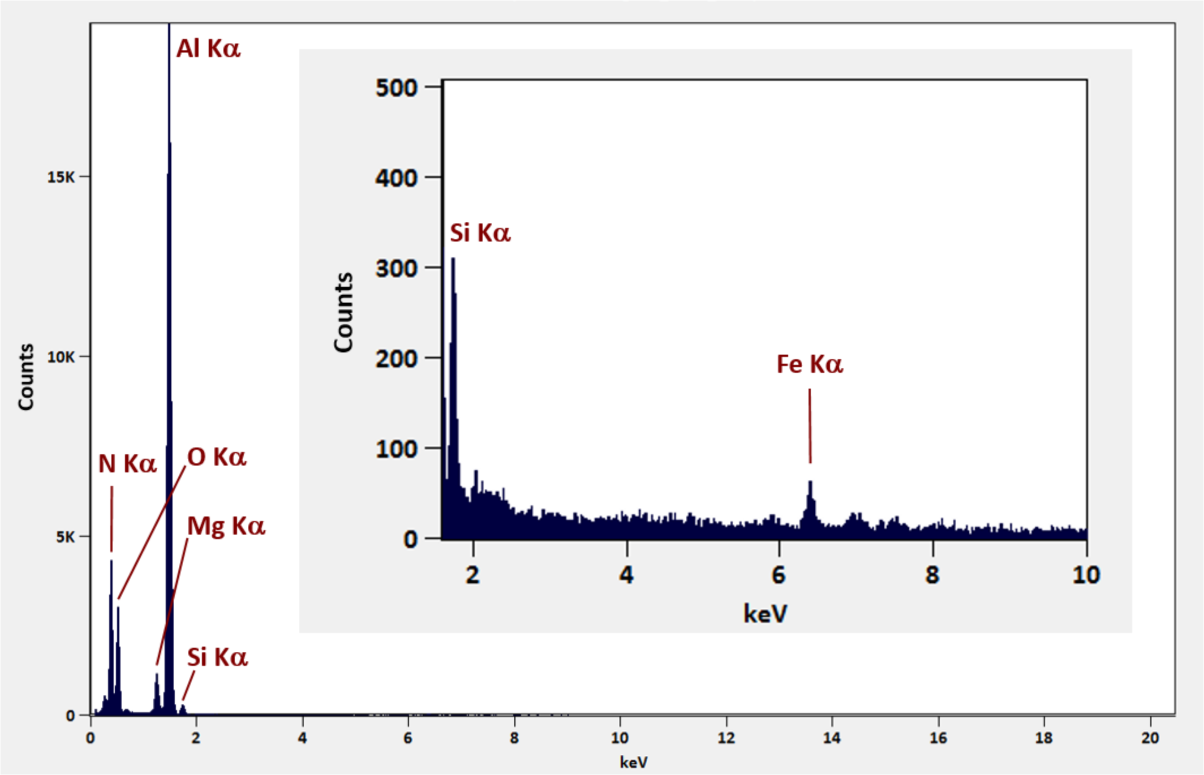

Figure S5: EDS spectrum used for the EDS mapping shown above. In addition to the N Kα, O Kα, Mg Kα, Al Kα, Si Kα peaks, Fe Kα peak can be clearly identified in the enlarged panel.

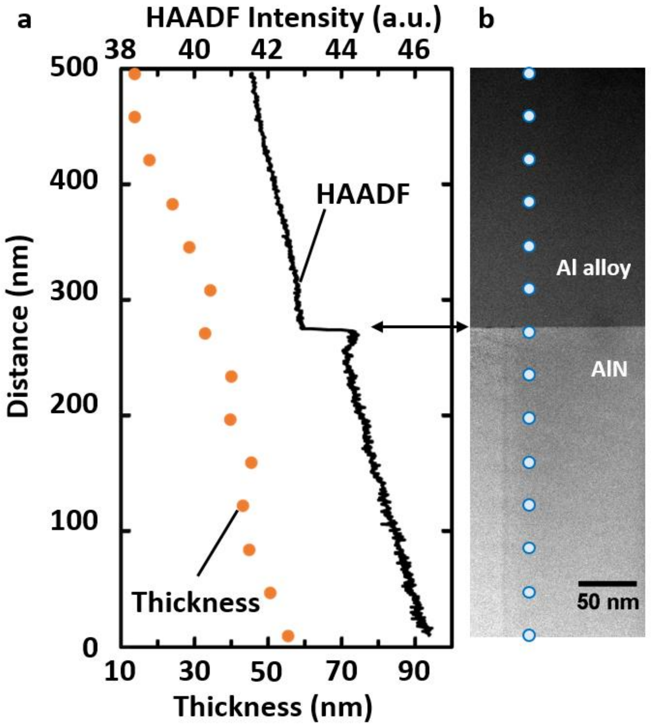

Figure S6: Specimen thickness and HAADF intensity profiles across the Al alloy/AlN interface. (**a**) Specimen thickness estimated using the electron energy loss spectroscopic (EELS) log-ratio method (Enfinium; Gatan, Inc.) and HAADF intensity profile (shown in (**b**)). EELS profiles were obtained with dwell time of 2 ms at each position shown in (**b**). For EELS acquisition, we use the energy window from zero to 900 eV with an energy dispersion of 0.5 eV/channel, convergence half-angle of 22 mrad and collection semi-angle of 25 mrad. The absolute thickness was calculated using effective atomic number of Al and AlN as the described in the literature[1]. (**b**) The HAADF image of the interface. The interface position is indicated by an arrow. When crossing the interface from the AlN side into Al alloy side, the intensity of HAADF suddenly drops, but the sample thickness monotonically decreases across the interface. This indicates there is no severe preferential ion milling at the interface regions.

Reference

1. Malis, T., Cheng, S.C., & Egerton, R.F. EELS log-ratio technique for specimen thickness measurement in the TEM. *J. Electron Microscope Technique* **8**, (1988) 193-200.
